# A Comparative Study for Detection of* EGFR* Mutations in Plasma Cell-Free DNA in Korean Clinical Diagnostic Laboratories

**DOI:** 10.1155/2018/7392419

**Published:** 2018-05-08

**Authors:** Yoonjung Kim, Saeam Shin, Kyung-A Lee

**Affiliations:** ^1^Department of Laboratory Medicine, Yonsei University College of Medicine, Seoul, Republic of Korea; ^2^Department of Laboratory Medicine, Hallym University College of Medicine, Kangnam Sacred Heart Hospital, Seoul, Republic of Korea

## Abstract

Liquid biopsies to genotype the epidermal growth factor receptor* (EGFR)* for targeted therapy have been implemented in clinical decision-making in the field of lung cancer, but harmonization of detection methods is still scarce among clinical laboratories. We performed a pilot external quality assurance (EQA) scheme to harmonize circulating tumor DNA testing among laboratories. For EQA, we created materials containing different levels of spiked cell-free DNA (cfDNA) in normal plasma. The limit of detection (LOD) of the cobas®* EGFR* Mutation Test v2 (Roche Molecular Systems) was also evaluated. From November 2016 to June 2017, seven clinical diagnostic laboratories participated in the EQA program. The majority (98.94%) of results obtained using the cobas assay and next-generation sequencing (NGS) were acceptable. Quantitative results from the cobas assay were positively correlated with allele frequencies derived from digital droplet PCR measurements and showed good reproducibility among laboratories. The LOD of the cobas assay was 5~27 copies/mL for p.E746_A750del (exon 19 deletion), 35~70 copies/mL for p.L858R, 18~36 copies/mL for p.T790M, and 15~31 copies/mL for p.A767_V769dup (exon 20 insertion). Deep sequencing of materials (>100,000X depth of coverage) resulted in detection of low-level targets present at frequencies of 0.06~0.13%. Our results indicate that the cobas assay is a reliable and rapid method for detecting* EGFR* mutations in plasma cfDNA. Careful interpretation is particularly important for p.T790M detection in the setting of relapse. Individual laboratories should optimize NGS performance to maximize clinical utility.

## 1. Introduction

Circulating tumor DNA (ctDNA) carries the same molecular alterations as the tumor itself and can be used to select treatment, assess the emergence of drug resistance, and monitor lung cancer patients in routine clinical practice [1]. The fraction of tumor-derived cell-free DNA (cfDNA) in blood plasma varies according to tumor stage, tumor burden, vascularization of the tumor, biological features of the tumor such as apoptotic rate, and the metastatic potential of the cancer cells [2]. Tumor-derived ctDNA often represents a small percentage of the total cfDNA and can be present at allele fractions as low as 0.01% [3]. Therefore, highly sensitive methodologies have been developed to detect low abundance epidermal growth factor receptor* (EGFR)* mutations, including p.T790M, from cfDNA in non-small cell lung cancer (NSCLC) patients, although the sensitivities and specificities of the methods vary [4, 5].

A sensitive method is needed to detect the p.T790M mutation in relapsed tumors because of tumor heterogeneity [6]. Recently, several* in vitro* diagnostics (IVD) have been approved by the European Medicines Agency (EMA) and the US Food and Drug Administration (FDA) for detecting* EGFR* mutations in plasma [7]. To ensure optimal quality molecular testing, clinical laboratories should evaluate the technical performance of ctDNA testing according to the standards from formal accreditation bodies, such as Clinical Laboratory Improvement Amendments (CLIA) and ISO 15189 [8, 9]. External quality assessment (EQA) is a way to standardize interlaboratory results and to monitor and improve testing processes across laboratories [10].

In this study, we designed EQA materials to evaluate the limit of detection (LOD) of the cobas* EGFR* Mutation Test v2 (Roche Molecular Systems, Inc., Branchburg, NJ, USA) and Oncomine Lung cfDNA Assay (Thermo Fisher Scientific, Waltham, MA, USA). We also implemented a pilot EQA scheme to assess the interlaboratory comparability of plasma* EGFR* testing results.

## 2. Materials and Methods

### 2.1. Preparation of EQA Materials

#### 2.1.1. Pooled Normal Human Plasma (NHP) Preparation

The workflow of the study process is shown in Supplementary Figure S1. Pooled normal human K2 EDTA plasma (NHP) was prepared using residual specimens from healthy individuals and was separated within 4 hours of collection. Negativity for* EGFR* mutation of 2 mL NHP was confirmed using the cobas* EGFR* Mutation Test v2 (Roche Molecular Systems, Inc.). cfDNA was extracted from 2 mL NHP using the MagMAX™ Cell-Free DNA Isolation Kit (Thermo Fisher Scientific) according to the manufacturer's instructions. cfDNA concentration and fragment size distribution were assessed using a 2200 TapeStation Instrument (Agilent Technologies, Santa Clara, CA, USA) with the Agilent D1000 ScreenTape System. Average fragment size was 185 bp, and average cfDNA concentration was 0.106 ng/*μ*L. We calculated that there were 1,162 wild-type copies per 2 mL NHP.

#### 2.1.2. EQA Material to Evaluate Assay Sensitivity

To prepare spiked materials with known mutant allele frequencies and mutant DNA copies, Multiplex I cfDNA Reference Standards (Horizon Discovery, Cambridge, UK) were purchased. This set is composed of wild-type cfDNA with mutant allele frequencies of 5%, 1%, 0.1%, and 0%. For each reference standard, copies per *μ*L of wild-type and mutant DNA were measured using digital droplet PCR and compared with the values provided by the manufacturer (Supplementary Table S1).

Four levels (levels 1 to 4) of spiked NHP (2 mL per sample) were prepared to determine the LOD of the detection assays. Intended mutant allele frequencies were 5%, 2.5%, 1%, and 0.1%, with 4 to 760 mutant copies in a background of about 10,000~16,000 wild-type copies in a spiked NPH samples, depending on the mutation. cfDNA was extracted from 2 mL spiked NHP using MagMAX Cell-Free DNA Isolation Kit (Thermo Fisher Scientific). Concentration and fragment size distribution of cfDNA were assessed using a 2200 TapeStation Instrument (Agilent Technologies). Average fragment size was about 190 bp, and the range of cfDNA was 60.42 ng to 80.18 ng. Details are provided in Supplementary Table S1.

#### 2.1.3. EQA Material to Evaluate Assay Precision

Genomic DNA from cell lines harboring mutations of the* EGFR* gene including p.T790M (HD258), p.L858R (HD254), and p.E746_A750del (HD251) were purchased from Horizon Discovery. To simulate the size distribution of cfDNA, each genomic DNA was fragmented to about 180~190 bp by sonication using a Covaris M220 (330 sec, 0.2% duty, 50 peak incident power, and 200 cycles/burst; Covaris Inc., Woburn, MA, USA) (Supplementary Table S2 and Supplementary Figure S2). Three spiked NHPs (1 mL per sample) were prepared to evaluate assay precision. Intended mutant allele frequencies were about 5%, with 1,221 to 1,503 mutant copies in a background of nearly 23,000~30,000 wild-type copies in spiked NPH samples, depending on the mutation. cfDNA was extracted from 1 mL spiked NHP using MagMAX Cell-Free DNA Isolation Kit (Thermo Fisher Scientific). Concentration and fragment size distribution of cfDNA were assessed using a 2200 TapeStation Instrument (Agilent Technologies instructions). Average size was 190 bp, and the cfDNA concentration (ng/mL) was 74.5~80.2. Details are provided in Supplementary Table S2. Spiked NHPs were frozen at <−70°C until genotyping.

### 2.2. Validation of EQA Material for LOD Evaluation

#### 2.2.1. cfDNA Extraction from Spiked NHP Samples

cfDNA was extracted from spiked NHP samples using MagMAX Cell-Free DNA Isolation Kit (Thermo Fisher Scientific) for next-generation sequencing and the cobas cfDNA Sample Preparation Kit (Roche Molecular Systems, Inc.) for the cobas* EGFR* assay. cfDNA concentration and purity were assessed using a NanoDrop 1000 spectrometer (Thermo Scientific, Waltham, MA, USA) and a Qubit 2.0 Fluorometer (Life Technologies, Grand Island, NY, USA) using the Qubit™ dsDNA HS Assay Kit. Size and amount of DNA fragments were assessed using a 2200 TapeStation Instrument (Agilent Technologies) with the Agilent D1000 ScreenTape System (Agilent Technologies).

#### 2.2.2. Evaluation of EQA Material Using Next-Generation Sequencing

For next-generation sequencing (NGS), a library was prepared using the Oncomine Lung cfDNA Assay and quantitated using qPCR. Emulsion PCR was performed using the Ion Chef System and Ion AmpliSeq IC 200 Kit (all Thermo Fisher Scientific). Barcoded libraries generated from 20 ng DNA per sample were loaded on an Ion 530 chip and sequenced on the Ion S5 XL System using Ion 520 and Ion 530 Kit-Chef (all Thermo Fisher Scientific). Alignment to the hg19 human reference genome and variant calling were performed using Torrent Suite™ software (Thermo Fisher Scientific). Variant annotation was performed using Ion Reporter™ Software 5.2 (Thermo Fisher Scientific). Torrent Suite software provides molecular coverage depth as well as read coverage depth at target bases to increase detection sensitivity for low-frequency variants [11, 12]. The manufacturer recommends a median read coverage >25,000X and median molecular coverage >2,500X to detect a variant with an allele frequency of 0.1%. Measured allele frequency (%) was calculated as mutant coverage depth divided by total coverage depth.

#### 2.2.3. Evaluation of LOD Material Using Real-Time PCR

For the cobas* EGFR* assay, 75 *μ*L DNA from each sample was loaded into three reaction wells (25 *μ*L DNA per well). Amplification and detection were performed using the cobas z 480 analyzer (Roche Molecular Systems, Inc.). Data were interpreted by the cobas z 480 software if positive and negative controls showed valid results. When a mutation was detected, semiquantitative index (SQI) values for each mutation are reported automatically by the software using the observed threshold cycle for the target mutation. The SQI was developed to measure trends in the amount of mutant cfDNA in a patient [13].

**Figure 1:**
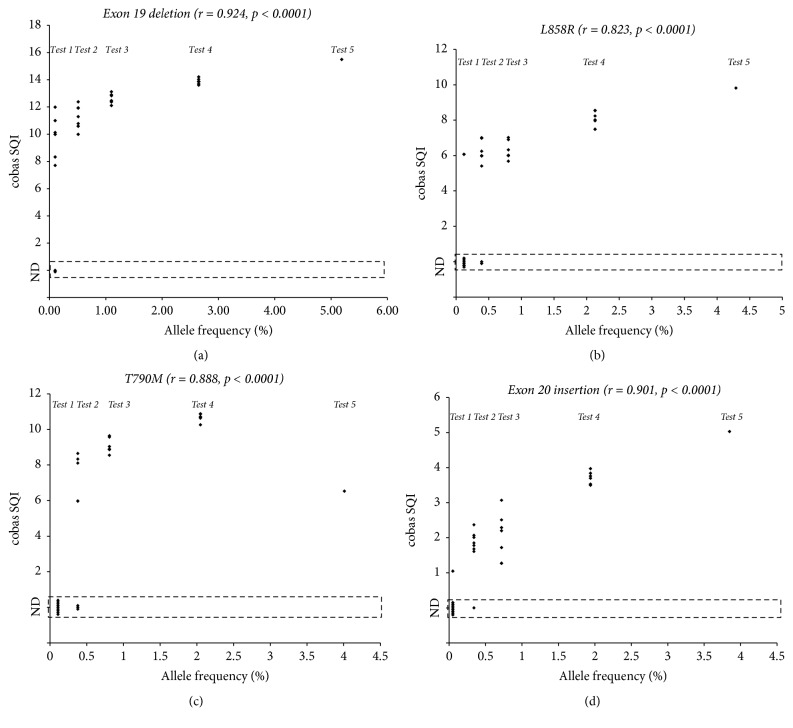
Analytical sensitivity of the cobas* EGFR* assay for NHP spiked with five different levels of mutant cfDNA. Copy numbers and frequencies of mutant alleles are provided in Supplementary Table S3. Dots in the figure represent negative measurement (ND) or the SQI value of the positive measurement for (a) p.E746_A750del (exon 19 deletion), (b) p.L858R, (c) p.T790M, and (d) p.A767_V769dup (exon 20 insertion) mutations.

The analytical performance of the cobas* EGFR* assay was additionally evaluated using five NHP samples spiked with different mutant allele frequencies that were made using Multiplex I cfDNA Reference Standards (Horizon Discovery, Cambridge, UK). These test samples had expected mutant allele frequencies of 3.85~5.19%, 1.94~2.65%, 0.72~1.10%, 0.34~0.51%, and 0.05~0.12% (Supplementary Table and Figure 1).

### 2.3. Distribution of EQA Material, Data Collection, and Analysis

Each laboratory director requested the amount of EQA material needed according to the number of methods planned for plasma* EGFR* testing. The number of reactions per test method among participating laboratories was 13. The EQA material set comprised four samples (2 mL, levels 1 to 4) with different mutant allele frequencies (5.0%, 2.5%, 1%, and 0.1%) for LOD analysis, and three samples with different* EGFR* mutations were also provided (1 mL per a reaction, P-1 to P-3).

For each test method, one EQA material set with 2 mL of LOD materials (levels 1 to 4) and 3 mL precision materials (P-1 to P-3) were distributed to each participating laboratory. Materials in barcoded K2 EDTA tubes were shipped to laboratories at 4°C along with a results datasheet to record qualitative results (detected or not detected) and quantitative results (SQI value from the cobas* EGFR* assay and total/mutant coverage depth from next-generation sequencing) for each mutation. Submitted qualitative results were evaluated as acceptable (positive for expected mutations or negative for unexpected mutations) or unacceptable (negative for expected mutations or positive for unexpected mutations), according to the manufactured and validated target mutations in this study (Table 1 and Supplementary Table S2). LOD level 4 material, which had an expected mutant allele frequency of 0.05~0.12%, was not graded. For the cobas assay, the mean, standard deviation, coefficient of variation (CV), median value, minimum value, and maximum value of data from the peer group and the standard deviation index of the data from the laboratory were provided in the evaluation reports.

### 2.4. Statistical Analysis

Statistical analysis was performed using SPSS Statistics version 24.0.0 (IBM Corp., Armonk, NY, USA). Correlations between SQI from cobas assay and mutant allele frequency were analyzed using Spearman rank-correlation test. All *p* values were two-sided, and values less than 0.05 were considered significant.

## 3. Results

### 3.1. Validation of EQA Materials

EQA materials for LOD evaluation were validated using the Oncomine Lung cfDNA Assay and cobas* EGFR *Mutation Test (Table 1). In a deep sequencing run, all four quality control samples were sequenced with high median coverage depth of more than 86,321X. All target bases showed adequate coverage (>500X). There was sufficient coverage at all target mutations to detect variants with allele frequencies of 0.1% (2,236~4,439X). All targeted mutations were called in levels 1–4 materials at similar allele frequencies to what were expected. cobas assay detected not only all target mutations in levels 1–3 materials but also p.L858R (0.12% allele frequency) and p.E746_A750del (exon 19 deletion; 0.10% allele frequency) in level 4 material.

### 3.2. Analytical Sensitivity of Real-Time PCR

Analytical sensitivity (LOD) of the cobas* EGFR* assay was assessed using five NHP samples spiked with different amounts of mutated targets (Figure 1 and Supplementary Table S3). Seven of nine cobas assays (77.8%) detected the exon 19 deletion in Test 5 material. All eight measurements of Test 4 material detected the exon 19 deletion. Six (75%), five (62.5%), and seven (87.5%) of eight measurements detected p.L858R, p.T790M, and p.A767_V769dup (exon 20 insertion) mutations in the Test 4 material, respectively. Therefore, the LODs of the cobas* EGFR* assay were determined to be 5~27 copies/mL for exon 19 deletion (0.1~0.5% allele frequency), 35~70 copies/mL for p.L858R (0.4~0.8% allele frequency), 18~36 copies/mL for p.T790M (0.4~0.8% allele frequency), and 15~31 copies/mL for exon 20 insertion (0.3~0.7% allele frequency). For all mutations, SQI values from the cobas assay exhibited a strong positive correlation with the expected mutant allele frequencies derived from digital droplet PCR measurements (Spearman rank-correlation coefficient, 0.823~0.924; *p* < 0.0001).

### 3.3. Pilot EQA Scheme

In November 2016, seven clinical laboratories that perform plasma* EGFR* molecular testing were recruited for the pilot EQA scheme (Table 2). In April 2017, EQA materials were made and distributed to each laboratory. A month after distributing the EQA materials, all results were emailed from each laboratory to an organizing director. In June 2017, evaluation reports were distributed to participating laboratories. Plasma* EGFR* testing was performed using an IVD assay and two laboratory-developed tests based on NGS: the cobas* EGFR* assay (*n* = 7), the Oncomine Lung cfDNA Assay on the Ion S5 XL (*n* = 1), and the QIAGEN GeneRead QIAact Actionable Insights Tumor Panel (QIAGEN, Hilden, Germany) on the GeneReader Platform (QIAGEN) (*n* = 1). There were two unacceptable results for NGS of LOD level 3 material (Tables 3 and 5).

### 3.4. Interlaboratory Comparability of Real-Time PCR Results

All results obtained using the cobas assay were concordant except for detection of* EGFR* exon 19 deletion and p.L858R in LOD level 4 material. Among seven laboratories, only six laboratories had a positive result for exon 19 deletion (detection rate 85.7%) and one laboratory had a positive result for p.L858R (detection rate 14.8%) in level 4 material. p.T790M and exon 20 insertion mutations were not detected in LOD level 4 material by any of the laboratories. The precision of SQI is summarized in Table 4. The cobas assay generally showed good reproducibility with a CV between 1.29% and 7.35% for target mutations. However, for p.T790M and exon 20 insertion, the CV for level 3 and/or level 4 materials (13.1% ~30.98%) was poorer than for the other mutations.

### 3.5. Interlaboratory Comparison of Next-Generation Sequencing Results

Mutant allele frequencies were calculated from the submitted depth of coverage data from NGS (Table 5). Results from two laboratories were consistent with the expected mutant allele frequencies calculated from absolute allele frequencies measured using digital droplet PCR. In laboratory F (S5XL + Oncomine Lung cfDNA Assay), all expected mutations were detected in level 3 material. Exon 19 deletion and exon 20 insertion mutations were detected at 0.15% (total read coverage depth of 73,836X) and 0.23% (total read coverage depth of 49,234X) in level 4 material, respectively. However, p.T790M and p.L858R mutations were not detected, despite the fact that total read coverage depth was not lower for these loci than other loci (65,455X for p.T790M and 70,849X for p.L858R). In laboratory C (GeneReader + QIAGEN GeneRead QIAact Actionable Insights Tumor Panel), p.T790M and exon 20 insertion mutations were not detected in level 3 material (unacceptable result). None of the four target mutations were detected in level 4 material.

## 4. Discussion

In this study, we prepared and validated EQA material for* EGFR* mutation detection using cfDNA and evaluated the analytical sensitivity of the cobas* EGFR* assay. According to the package insert, the LOD of the cobas assay using sheared DNA with an average size of 220 bp is less than 0.1% (75 copies/mL for exon 19 deletion, 25 copies/mL for the exon 20 insertion, and 100 copies/mL for p.L858R and p.T790M). In the present study, we confirmed the LODs of the cobas assay for each target mutation. The analytical sensitivities of the cobas assay were not identical for the different target mutations, similar to previous reports [14, 15]. In our pilot EQA, the cobas assay showed a higher detection rate and lower imprecision for exon 19 deletion and p.L858R than for p.T790M and exon 20 insertion. Similarly, in laboratory F that used the Oncomine Lung cfDNA Assay, p.T790M and p.L858R were not detected, despite adequate depth of coverage of the target site compared to other loci. This difference in assay performance according to target mutation might be due to the assay design and characteristics of the target regions [14, 15]. This finding is an important issue for detection of p.T790M in patients who show evidence of tumor progression after prior EGFR-tyrosine kinase inhibitor (TKI) therapy. Previous studies reported that it is challenging to detect the p.T790M mutation in patients with acquired resistance to prior EGFR-TKI therapy due to genomic heterogeneity [16, 17]. Therefore, caution is warranted in the setting of tumor relapse, and additional efforts should be made to optimize the experimental conditions to increase the sensitivity of p.T790M detection.

In our pilot EQA, participating laboratories performed one IVD assay (cobas* EGFR* assay) and two laboratory-developed tests based on NGS. The cobas assay showed reliable and robust test performance in all laboratories. SQI showed a positive correlation with mutant allele frequency derived from digital droplet PCR measurements. This finding is consistent with that of a previous study that evaluated clinical samples with NGS and the cobas assay [13]. Moreover, SQI from the cobas assay was reproducible among laboratories in our pilot EQA. Therefore, SQI could be useful for patient monitoring. About 3 hours of processing time is required for DNA extraction, PCR amplification, and detection in the cobas assay. Thus, this assay can be used for rapid and reliable plasma ctDNA analysis in clinical diagnostic laboratories.

A limitation of this study is the small number of laboratories that participated, especially laboratories performing NGS. It was unclear whether unacceptable responses were due to the performance of specific NGS methods or the laboratory. However, coverage depth results from two laboratories indicate that more read coverage depth is required to detect low-frequency variants in samples. In our validation experiment using the Oncomine Lung cfDNA Assay, all mutations were detected in level 4 material when the coverage depth was more than 100,000X. Our data and previous reports indicate that high coverage depth is essential to improve the detection of low-level targets [18, 19].

Another issue related to NGS is assay turn-around time (TAT). TAT for* EGFR* testing for NSCLC patients is important for drug selection. NGS generally requires more time than IVD, although it differs depending on batch constitution and the platform used. The two laboratories that performed NGS also used an IVD assay. The main advantage of NGS over IVD is scalability for type of mutation and target gene. Using NGS, rare (e.g., the p.C797S resistance mutation [20]) or novel mutations in* EGFR*, as well as other genes, can be identified [21]. Moreover, advanced NGS technology enables detection of not only point mutation but also gene fusions and amplifications [22, 23].

In the era of companion diagnostics, more mutations will be used as predictive markers to determine patient eligibility for molecular-targeted therapies. As a result, rigorous quality controls to avoid inappropriate patient treatment will become increasingly important in clinical diagnostic laboratories. EQA is critical for quality assurance and continuous improvement of individual laboratory performance [9]. Recently, Haselmann et al. have reported EQA scheme for ctDNA analysis of* KRAS* and* BRAF* genotyping, using mutant allele frequency of 0%, 5%, and 10% samples [24]. Digital approaches revealed no error rate, although Sanger sequencing revealed very high error rate around 20%. The authors suggested that method sensitivity correlates with diagnostic accuracy. Another EQA report for blood based* EGFR* p.T790M testing included pyrosequencing, digital PCR, and several allele-specific PCR platforms, using four levels of spiked materials [25]. Although we used limited number of methods of two NGS and one IVD platform, we suggested more delicate means of EQA workflow tailored to ctDNA testing, using strictly designed low-level materials to assess assay sensitivity and precision in individual laboratories. Larger trial including more genotyping platforms including digital PCR with our sample preparation protocol is worthy of further investigation. In our pilot EQA, we used spiked ctDNA samples, which will facilitate standardization and improvement of ctDNA testing practices in clinical diagnostic laboratories.

## Figures and Tables

**Table 1:** Validation of external quality assurance material using Oncomine Lung cfDNA Assay and cobas *EGFR* Mutation Test.

EQA materials	*EGFR* variant	Expected mutant allele frequency (%)	Oncomine Lung cfDNA Assay									SQI from cobas *EGFR* mutation test
			Number of mapped reads	Percentage of reads on target (%)	Median base coverage depth	Uniformity of base coverage (%)	Target base coverage at 500X (%)	Molecular coverage depth, total/mutant	Measured allele frequency (%)^a^	Read coverage depth, total/mutant	Measured allele frequency (%)^b^	
Level 1	p.L858R	4.29	3376397	89.72	91374	100	100	2999/53	1.77	151533/2489	1.64	9.82
	p.E746_A750del	5.19						4026/118	2.93	88048/1523	1.73	15.49
	p.T790M	4.01						3674/117	3.18	97546/3212	3.29	11.93
	p.A767_V769dup	3.85						3834/122	3.18	58931/2214	3.76	5.06
Level 2	p.L858R	2.13	4144029	89.28	108108	100	100	2843/56	1.97	160148/3418	2.13	7.49
	p.E746_A750del	2.65						4174/90	2.16	103525/1216	1.17	13.88
	p.T790M	2.05						3942/82	2.08	109822/2170	1.98	10.71
	p.A767_V769dup	1.94						4349/74	1.70	77146/1661	2.15	3.5
Level 3	p.L858R	0.80	3410157	87.94	86321	100	100	2236/6	0.27	122767/477	0.39	6.67
	p.E746_A750del	1.10						3533/39	1.10	88048/523	0.59	12.9
	p.T790M	0.81						3269/31	0.95	92814/927	1.00	9.28
	p.A767_V769dup	0.72						3655/21	0.57	63023/462	0.73	2.68
Level 4	p.L858R	0.12	4252130	88.17	108397	100	100	2692/2	0.07	159720/152	0.10	6.07
	p.E746_A750del	0.10						3880/4	0.10	110976/117	0.11	9.99
	p.T790M	0.11						3770/5	0.13	117037/149	0.13	ND
	p.A767_V769dup	0.05						3877/4	0.10	79321/198	0.25	ND

^a^Allele frequency (%) = mutant molecular coverage depth/total molecular coverage depth. ^b^Allele frequency (%) = mutant read coverage depth/total read coverage depth. EQA, external quality assurance; SQI, semiquantitative index; ND, not detected.

**Table 2:** Plasma *EGFR* genotyping methods by laboratories participating in pilot external quality assurance.

Laboratories	In vitro diagnostics	Laboratory-developed tests	
	cobas z 480 + cobas *EGFR* Mutation Test v2	S5XL + Oncomine Lung cfDNA Assay	GeneReader + QIAGEN GeneRead QIAact Actionable Insights Tumor Panel
A	O		
B	O		
C	O		O
D	O		
E	O		
F	O	O	
G	O		

**Table 3:** Unacceptable response rate in pilot external quality assurance scheme.

Material^a^	*EGFR* variant	Acceptable response/total response (*n*)									Unacceptable response/total response (*n*)	Unacceptable response rate (%)
		cobas *EGFR *Mutation Test (7 laboratories)							NGS (2 laboratories)			
		A	B	C	D	E	F	G	C	F		
Level 1	p.L858R; p.E746_ A750del; p.T790M; p.A767_V769dup	4/4	4/4	4/4	4/4	4/4	4/4	4/4	4/4	4/4	0/36	0.0
Level 2		4/4	4/4	4/4	4/4	4/4	4/4	4/4	4/4	4/4	0/36	0.0
Level 3		4/4	4/4	4/4	4/4	4/4	4/4	4/4	2/4	4/4	2/36	5.6
Level 4		-/4	-/4	-/4	-/4	-/4	-/4	-/4	-/4	-/4	Not graded^b^	-
P-1	p.T790M	3/3	3/3	3/3	3/3	3/3	3/3	3/3	3/3	3/3	0/26^c^	0.0
P-2	p.L858R	3/3	3/3	3/3	3/3	3/3	3/3	3/3	3/3	3/3	0/27	0.0
P-3	p.E746_A750del	3/3	3/3	3/3	3/3	3/3	3/3	3/3	3/3	3/3	0/27	0.0

^a^Level 1~level 4 materials were prepared for a limit of detection and P-1~P-3 materials were prepared for precision evaluation. P-1~P-3 materials were split and run on three replications. ^b^For cobas assay, p.L858R mutation was detected in one among seven laboratories (14.3%), and p.E746_ A750del (exon 19 deletion) mutation was detected in six among seven laboratories (85.7%). For NGS, exon 19 deletion and p.A767_V769dup (exon 20 insertion) were detected in laboratory F. ^c^One measurement of P-1 material showed an invalid result in laboratory C. NGS, next-generation sequencing.

**Table 4:** The performance of interlaboratory comparison samples using the cobas *EGFR* Mutation Assay.

Sample	p.L858R					p.E746_A750del (exon 19 deletion)					p.T790M					p.A767_V769dup (exon 20 insertion)				
	Expected allele frequency (%)^a^	Copies of mutant DNA (/*μ*L)^a^	Mean	SD	CV (%)	Expected allele frequency (%)^a^	Copies of mutant DNA (/*μ*L)^a^	Mean	SD	CV (%)	Expected allele frequency (%)^a^	Copies of mutant DNA (/*μ*L)^a^	Mean	SD	CV (%)	Expected allele frequency (%)^a^	Copies of mutant DNA (/*μ*L)^a^	Mean	SD	CV (%)
Level 1	4.29	760	9.91	0.61	6.16	5.19	600	15.14	0.27	1.79	4.01	420	11.28	0.5	4.43	3.85	362	4.52	0.25	5.57
Level 2	4.02	380	8.65	0.5	5.76	4.72	300	14.24	0.18	1.29	3.61	210	10.25	0.59	5.74	3.42	181	3.56	0.47	13.1
Level 3	0.8	140	6.93	0.47	6.79	1.1	108	12.03	0.56	4.63	0.81	72	7.4	1.78	24.08	0.72	62	2.07	0.64	30.98
Level 4	0.12	20	-	-	-	0.1	10	8.91	0.63	7.11	0.11	10	-	-	-	0.05	4	-	-	-
Precision materials (P-1~P-3)^b^	3.0	NP	9.99	0.46	4.61	3.0	NP	15.2	0.27	1.78	3.0	NP	11.03	0.31	2.83	-	-	-	-	-
	3.0	NP	9.77	0.53	5.46	3.0	NP	15.2	0.3	1.95	3.0	NP	11.22	0.49	4.38	-	-	-	-	-
	3.0	NP	9.75	0.72	7.35	3.0	NP	15.22	0.32	2.09	3.0	NP	11.19	0.59	5.24	-	-	-	-	-

^a^Mutant allelic frequency and copies of mutant DNA in materials were adjusted using next-generation sequencing result from information provided by the manufacturer of reference standards. ^b^Only positive results from precision materials were summarized in the table. SQI, semiquantitative index; SD, standard deviation; CV, coefficient of variation; NP, not provided.

**Table 5:** Comparison of mutant allelic frequency from two laboratories using different next-generation sequencing platforms.

Sample	p.L858R				p.E746_A750del (exon 19 deletion)				p.T790M				p.A767_V769dup (exon 20 insertion)			
	Expected mutant allele frequency (%)	Lab F		Lab C	Expected mutant allele frequency (%)	Lab F		Lab C	Expected mutant allele frequency (%)	Lab F		Lab C	Expected mutant allele frequency (%)	Lab F		Lab C
		Molecular coverage depth, total/mutant (allele frequency, %)	Read coverage depth, total/mutant (allele frequency, %)	Read coverage depth, total/mutant (allele frequency, %)		Molecular coverage depth, total/mutant (allele frequency, %)	Read coverage depth, total/mutant (allele frequency, %)	Read coverage depth, total/mutant (allele frequency, %)		Molecular coverage depth, total/mutant (allele frequency, %)	Read coverage depth, total/mutant (allele frequency, %)	Read coverage depth, total/mutant (allele frequency, %)		Molecular coverage depth, total/mutant (allele frequency, %)	Read coverage depth, total/mutant (allele frequency, %)	Read coverage depth, total/mutant (allele frequency, %)
Level 1	4.29	1970/67 (3.40)	74088/2440 (3.29)	4594/171 (3.72)	5.19	3360/131 (3.90)	60991/1272 (2.09)	5819/231 (3.97)	4.01	3182/124 (3.90)	59366/2276 (3.83)	1717/71 (4.14)	3.85	3258/130 (3.99)	38689/1707 (4.41)	1091/30 (2.75)
Level 2	2.13	1755/41 (2.34)	70894/1707 (2.41)	4385/84 (1.92)	2.13	2855/65 (2.28)	61723/753 (1.22)	10001/130 (1.30)	2.05	2680/51 (1.90)	56596/983 (1.74)	2572/87 (3.38)	1.94	2670/53 (1.99)	37564/541 (2.37)	2341/24 (1.03)
Level 3	0.80	1912/15 (0.78)	107664/791 (0.73)	4681/52 (1.11)	1.10	3095/22 (0.71)	94144/378 (0.40)	9416/57 (0.61)	0.81	2902/26 (0.90)	84166/754 (0.90)	ND^a^	0.72	3667/21 (0.57)	57591/541 (0.94)	ND^a^
Level 4	0.12	1804/0 (ND)	70849/0 (ND)	ND^a^	0.10	3158/3 (0.09)	73836/114 (0.15)	ND^a^	0.11	2980/0 (ND)	65455/0 (ND)	ND^a^	0.05	3296/3 (0.09)	49234/115 (0.23)	ND^a^
Precision materials (P-1~P-3)^b^	3.00	2250/89 (3.96)	97519/3743 (3.84)	4067/148 (3.64)	3.00	2306/68 (2.95)	45162/1336 (2.96)	9157/250 (2.73)	3.00	3132/126 (4.02)	60226/1396 (2.32)	2131/111 (5.21)	-	-	-	-
	3.00	2259/94 (4.16)	124662/5042 (4.04)	6335/344 (5.43)	3.00	3326/114 (3.43)	110580/3431 (3.1)	8823/274 (3.11)	3.00	2980/112 (3.76)	88684/3164 (3.57)	2904/126 (4.34)	-	-	-	-
	3.00	2200/72 (3.27)	87890/2799 (3.18)	4638/139 (2.99)	3.00	3462/134 (3.87)	81882/1905 (2.33)	14136/242 (1.71)	3.00	3380/123 (3.64)	89937/3303 (3.67)	Invalid^c^	-	-	-	-

^a^Coverage depth was not provided by the laboratory. ^b^Only positive results from precision materials were summarized in the table. ^c^One measurement of P-1 material showed invalid result using next-generation sequencing from a laboratory. ND, not detected.

## Supplementary Materials

Supplementary Materials Supplementary Figure S1: workflow of the pilot external quality assurance scheme. Supplementary Figure S2: electropherograms of DNA sheared by ultrasonication and run on an Agilent D1000 ScreenTape System. Supplementary Table S1: external quality assurance material for evaluation of limit of detection. Supplementary Table S2: external quality assurance material for evaluation of precision. Supplementary Table S3: preparation material for performance evaluation.
