# Supplementary material for: A Comparative Study for Detection of EGFR Mutations in Plasma Cell-Free DNA in Korean Clinical Diagnostic Laboratories
Source: Biomed Res Int. 2018 May 8;2018:7392419. doi: 10.1155/2018/7392419 (PMC5964486; doi:10.1155/2018/7392419)
Supplement: Supplementary Materials — Supplementary Figure S1: workflow of the pilot external quality assurance scheme. Supplementary Figure S2: electropherograms of DNA sheared by ultrasonication and run on an Agilent D1000 ScreenTape System. Supplementary Table S1: external quality assurance material for evaluation of limit of detection. Supplementary Table S2: external quality assurance material for evaluation of precision. Supplementary Table S3: preparation material for performance evaluation. [file 7392419.f1.pdf]

Supplementary materials

Supplementary Figure S1. Workflow of the pilot external quality assurance scheme.

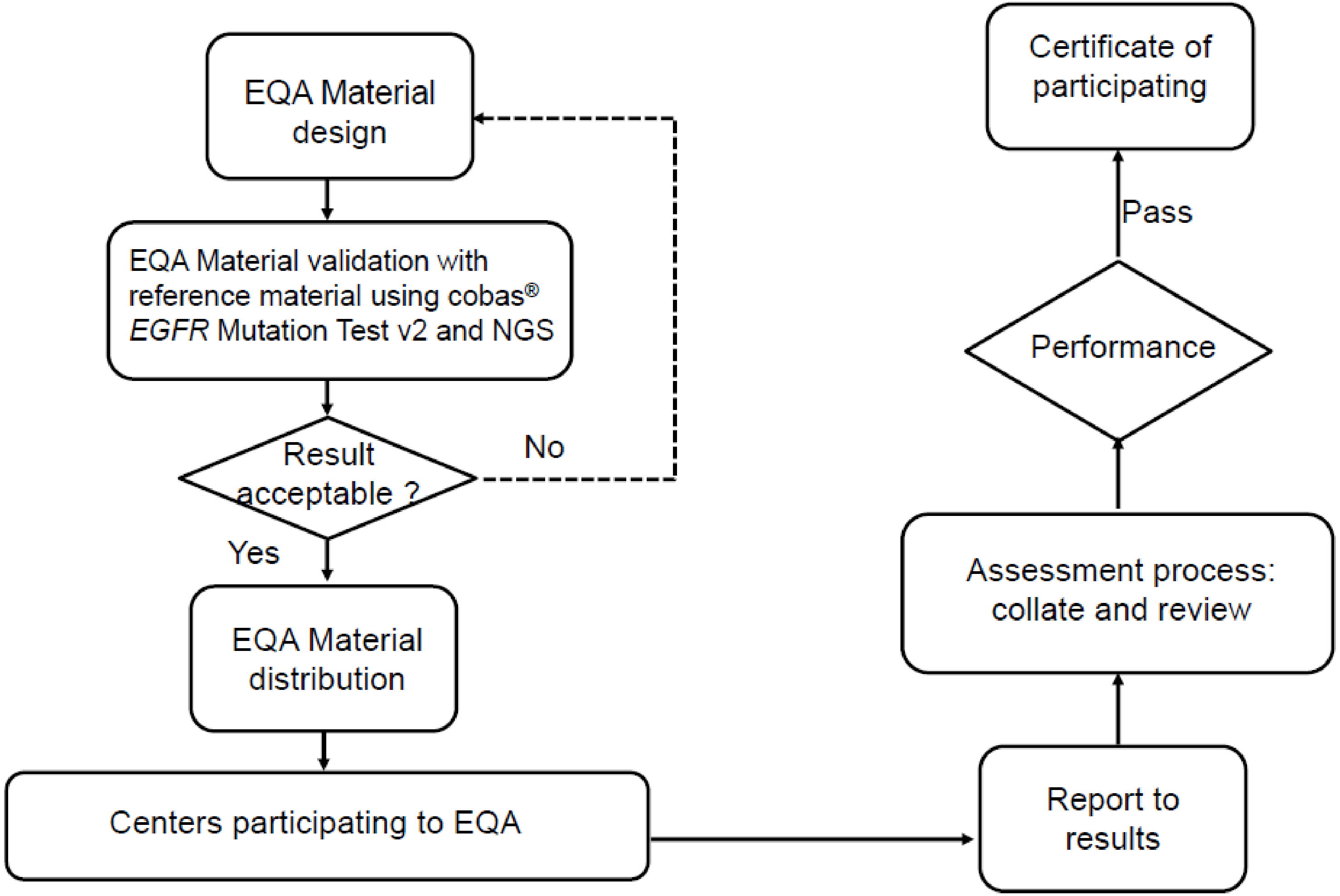

Supplementary Figure S2. Electropherograms of DNA sheared by ultrasonication and run on an Agilent D1000 ScreenTape System. Sheared genomic DNA from wild-type and (a) the *EGFR* p.T790M reference standard and (b) the *EGFR* p.L858R reference standard.

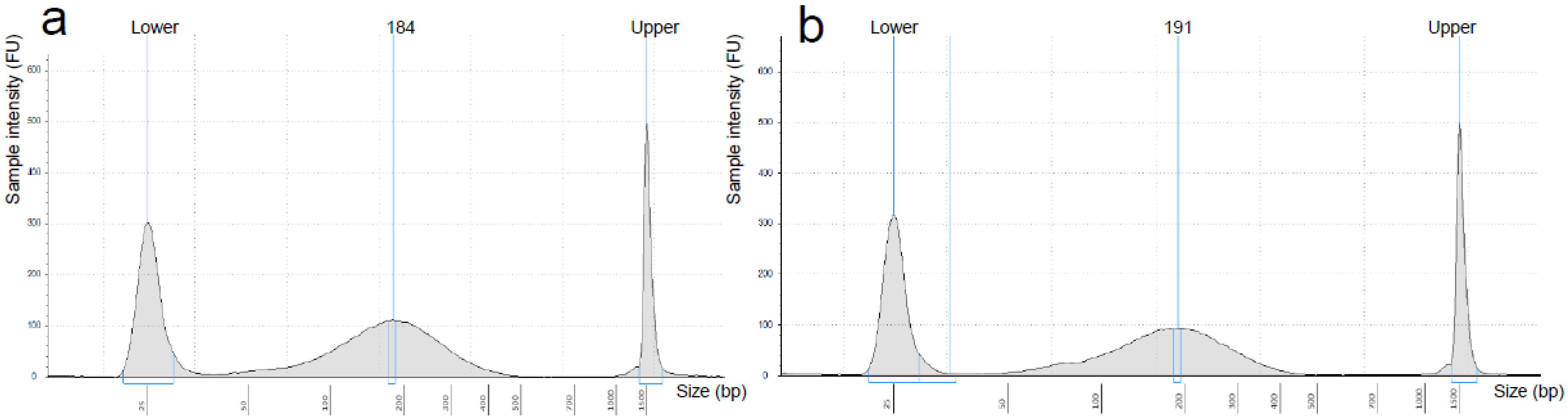

Supplementary Table S1. External quality assurance material for evaluation of limit of detection

EQA materials	Reference Materials	Gene	Variant	Allele frequency (%)[a]	Copies of wild type DNA per μL[a]	Copies of mutant DNA per μL[a]	Spiked NHP (2 mL)[b] of		
							Expected copies of wild type DNA[c]	Expected copies of mutant DNA	The total fragmented DNA (ng)[d]
Level 1	5% Multiplex I cfDNA Reference Standard (HD777)	*EGFR*	p.L858R	4.59	7904	380	16970	760	80.18
			p.E746_A750del	5.77	4896	300	10954	600	
			p.T790M	4.52	4440	210	10042	420	
			p.A767_V769dup	4.39	3944	181	9050	362	
Level 2	2.5% Spiked Standard (HD777: WT100% = 1 : 1)	*EGFR*	p.L858R	2.29	8140	190	17442	380	60.42
			p.E746_A750del	2.89	4936	150	11034	300	
			p.T790M	2.26	4428	105	10018	210	
			p.A767_V769dup	2.19	3988	91	9138	181	
Level 3	1% Multiplex I cfDNA Reference Standard (HD778)	*EGFR*	p.L858R	0.85	8144	70	17450	140	68.02
			p.E746_A750del	1.24	4296	54	9754	108	
			p.T790M	0.93	3848	36	8858	72	
			p.A767_V769dup	0.84	3672	31	8506	62	
Level 4	0.1% Multiplex I cfDNA Reference Standard (HD779)	*EGFR*	p.L858R	0.13	7824	10	16810	20	65.74
			p.E746_A750del	0.11	4408	5	9978	10	
			p.T790M	0.13	3896	5	8954	10	
			p.A767_V769dup	0.06	3616	2	8394	4	

[a]Mutant allele frequency, copy number values of wild type and mutant DNA measured using digital droplet PCR were provided from manufacturer.

[b]1 μL of standard DNA were mixed with 1 mL of NHP.

[c]1162 wide type copies per 2 mL NHP were added.

[d]cfDNA was extracted from 2 mL of spiked NHP using MagMAX™ Cell-Free DNA Isolation Kit (Thermo Fisher Scientific) and then the cfDNA concentration and the fragment size distribution assessed using a 2200 TapeStation Instrument (Agilent Technologies).

* Abbreviation: EQA, external quality assurance; NHP, normal human plasma.

Supplementary Table S2. External quality assurance material for evaluation of precision

EQA materials	Reference Materials	Expected allele frequency [a]	The spiked DNA[b] were consists of						The spiked NHP[e] (1 mL) of			
			gDNA from reference standard DNA (50ng/μL)	Wild type DNA (μL) 50ng/μL	Total DNA (ng)	Measured concentration of sheared DNA (ng/μL)[c]	Expected copies of wild type DNA per μL[d]	Expected copies of Mutant DNA per μL[d]	Expected copies of mutant DNA	Expected copies of wild type DNA[f]	Allele frequency (%)	Extracted total DNA (ng/mL)[g]
P-1	*EGFR* p.T790M Reference Standard (HD258)	50%	10 μL	90 μL	5000	33.1	480.0	9119.1	1295.9	25202.4	4.9	74.5
P-2	*EGFR* p.L858R Reference Standard (HD254)	50%	10 μL	90 μL	5000	31.2	452.4	8595.6	1221.5	23789.1	4.9	80.2
P-3	*EGFR* p.E746_ A750del Reference Standard (HD251)	50%	10 μL	90 μL	5000	38.4	556.8	10579.2	1503.4	29144.8	4.9	79.5

[a]Expected mutant allelic frequency were measured using digital droplet PCR were provided from manufacturer.

[b]Spiked DNA was fragmented to about 180 ~190 bp by sonication using a Covaris M220 (Covaris Inc.).

[c]The concentration of sheared DNA which was distributed between 100 bp and 300 bp was assessed using a 2200 TapeStation Instrument (Agilent Technologies).

[d]Copies per ng were 290.

[e]2.7 μL of spiked DNA were mixed with 1 mL of NHP.

[f]581 wild type copies per 1 mL NHP were added.

[g]cfDNA was extracted from 1 mL of spiked NHP using MagMAX™ Cell-Free DNA Isolation Kit (Thermo Fisher Scientific) and then the concentration of sheared DNA which was distributed between 100 bp and 300 bp was assessed using a 2200 TapeStation Instrument (Agilent Technologies).

* Abbreviation: EQA, external quality assurance; NHP, normal human plasma.

Supplementary Table S3. Preparation material for performance evaluation

Materials	Reference Materials	Gene	Variant	Allele frequency (%)[a]	Copies of wild type DNA per μL[a]	Copies of mutant DNA per μL[a]	Spiked NHP (2 mL)[b] of		
							Expected copies of wild type DNA[c]	Expected copies of mutant DNA	Expected mutant allele freqeuncy (%)
Test 1	5% Multiplex I cfDNA Reference Standard (HD777)	*EGFR*	p.L858R	4.59	7904	380	16970	760	4.29
			p.E746_ A750del	5.77	4896	300	10954	600	5.19
			p.T790M	4.52	4440	210	10042	420	4.01
			p.A767_V769dup	4.39	3944	181	9050	362	3.85
Test 2	2.5% Spiked Standard (HD777: WT100%=1:1)	*EGFR*	p.L858R	2.29	8140	190	17442	380	2.13
			p.E746_ A750del	2.89	4936	150	11034	300	2.65
			p.T790M	2.26	4428	105	10018	210	2.05
			p.A767_V769dup	2.19	3988	91	9138	181	1.94
Test 3	1% Multiplex I cfDNA Reference Standard (HD778)	*EGFR*	p.L858R	0.85	8144	70	17450	140	0.80
			p.E746_ A750del	1.24	4296	54	9754	108	1.10
			p.T790M	0.93	3848	36	8858	72	0.81
			p.A767_V769dup	0.84	3672	31	8506	62	0.72
Test 4	0.5% Spiked Standard (HD778: WT100%=1:1)	*EGFR*	p.L858R	0.43	8260	35	17682	70	0.39
			p.E746_ A750del	0.62	4636	27	10434	54	0.51
			p.T790M	0.46	4132	18	9426	36	0.38
			p.A767_V769dup	0.42	3852	15	8866	30	0.34
Test 5	0.1% Multiplex I cfDNA Reference Standard (HD779)	*EGFR*	p.L858R	0.13	7824	10	16810	20	0.12
			p.E746_ A750del	0.11	4408	5	9978	10	0.10
			p.T790M	0.13	3896	5	8954	10	0.11
			p.A767_V769dup	0.06	3616	2	8394	4	0.05

[a]Mutant allele frequency, copy number values of wild type and mutant DNA measured using digital droplet PCR were provided from manufacturer.

[b]1 μL of standard DNA were mixed with 1 mL of NHP.

[c]1162 wild type copies per 2 mL NHP were added.
